# A study on the nutritional characteristics of some plants and their effects on ruminal microbial fermentation and protozoa population

**DOI:** 10.1186/s13568-021-01338-x

**Published:** 2021-12-22

**Authors:** Mohsen Kazemi, Mohammad Mehdi Moheghi, Reza Tohidi

**Affiliations:** 1 Department of Animal Science, Faculty of Agriculture and Animal Science, University of Torbat-e Jam, Torbat-e Jam, Iran; 2 Department of Animal Science, Agriculture Faculty, Herat University, Herat, Afghanistan

**Keywords:** Plants, Ruminant, Nutritional potential, Protozoa, Culture medium

## Abstract

We designed this project to determine the nutritional potential and ruminal microbial fermentation properties of eight rangeland plants (*Dracocephalum moldavica* L., *Melissa officinalis* L., *Ruta graveolens* L., *Perovskia abrotanoides* Kar., *Cichorium intybus* L., *Borago officinalis* L., *Peganum harmala* L., and *Teucrium polium* L.) collected from the semi-arid region of Iran at two consecutive years (2019 and 2020) for ruminant diets. *Medicago sativa* as a common forage was also considered as control. We determined the chemical-mineral composition, buffering capacity, in vitro gas yield, ruminal fermentation, and protozoa population in a culture medium with the standard laboratory methods. A significant difference in chemical-mineral compounds was observed among the studied plants (*p* < 0.05). A lower crude protein range (6.28% for *Cichorium intybus* L. to 18.4% for *Melissa officinalis* L.) was observed rather than *Medicago sativa* (20.3%). The amount of calcium was highest in *Peganum harmala* L. (23.5–24.2 g/kg DM) and lowest in *Ruta graveolens* L. (1.15–1.25 g/kg DM). *Dracocephalum moldavica* L. exhibited the highest acid–base buffering capacity (235–242 mEq×10^−3^) among other plants. The highest decrease in total protozoa and other protozoan populations was observed when *Perovskia abrotanoides* Kar. was added to the culture medium. *Teucrium Polium* L. had the greatest potential gas yield and its total volatile fatty acid was comparable with *Medicago sativa*. It seems that eight plants are nutritionally suitable for partial replacement of the conventional plants such as *Medicago sativa* in diets of small ruminants, however dietary supplementation of *Peganum harmala* L. due to its alkaloids content should be done with caution.

## Introduction

In the semi-arid and arid regions of Iran, there is an intense shortage of suitable forages for animal production. Also, in recent years, the carrying capacity of natural pastures and nutritional value of the rangelands has been restricted due to severe droughts, improper over-grazing, and lack of a proper grazing program (Habib et al. 2016). The natural pastures in the mountainous region of Iran provide an essential forage resource for small ruminants that complement lowland livestock systems during the grazing period (Kazemi 2019). Torbat-e Jam mountainous rangelands consist of herbs, shrubs, grasses, and trees which often have suitable biodiversity. Small ruminants in this region often obtain their feedstuffs through grazing on native pastures. Several studies on the nutritive value of some rangeland plants as feed sources for ruminants were recently conducted in Iran (Kazemi 2019; Kazemi and Valizadeh 2019; Kazemi 2021). Despite the ecological importance as a habitat for biodiversity flora and fauna, the Iranian rangelands consist of different plants containing a nutritional potential that can meet the nutrient requirements of small ruminants (Kazemi 2019; Kazemi and Valizadeh 2019). The use of rangeland plants in diets of ruminants depends on their chemical/mineral compounds and nutrient digestibility. The chemical composition and ruminal microbial fermentation parameters of plants can be diverse because of differences in the plant variety, environmental and weather situations (Keim et al. 2018). For example, the in vitro dry matter degradability of some range plants varied from 711 to 828 g/kg DM among the plants collected in the semi-arid pastures of Torbat-e Jam, Iran. Although different range plants exist in Iran, less attention has been paid to their nutritional aspects worldwide. Therefore, the objective of this study was to collect samples of eight rangeland plants in two consecutive years to determine their chemical-mineral compounds and buffering capacity and test their effects on ruminal microbial fermentation parameters and protozoa populations in vitro.

## Material and methods

### Area and plants collection

The whole samples of eight plant species including *Dracocephalum moldavica* L., *Melissa officinalis* L., *Ruta graveolens* L., *Perovskia abrotanoides* Kar., *Cichorium intybus* L., *Borago officinalis* L., *Peganum harmala* L., and *Teucrium polium* L., were randomly collected during the flowering stage from mountain pastures of Torbat-e Jam (Mian Jam rural district, altitude approximately 1200 m above sea level with semi-arid weather) located in the province of Razavi Khorasan between 2019 and 2020 (June to August). *Medicago sativa* was also collected from the surrounding lands of studied plants during the flowering stage.

## Laboratory protocols

### Chemical composition

The whole samples related to each plant in each year were mixed, transferred to the laboratory, and then dried at 60 °C in a forced oven (Behdad Co., Iran) for 48 h. After that, the samples were milled through a 1 mm screen (Willey mill), transferred to the nylon bags, and preserved in a dark place until the subsequent experiments. We determined dry matter (DM) related to each plant by a forced oven at 105 °C until constant weight (AOAC 2005). The protocols suggested by AOAC (2005) were employed for ether extract (EE), ash, and crude protein (CP, Kjeldahl N × 6.25) concentrations. The concentrations of neutral detergent fiber (NDF), acid detergent lignin (ADL), and acid detergent fiber (ADF) were measured according to Ankom procedures (Ankom Technology 2005, 2006a, b).

### Mineral composition and buffering capacity

The mineral concentrations of samples (sodium, magnesium, calcium, potassium, iron, zinc, manganese, and cobalt) were measured using atomic absorption (SavantAA, GBC, Australia). The phosphorus content was determined by UV–Vis spectrophotometry. The suggested method by Jasaitis et al. (1987) was considered for buffering characteristics and pH determination (Table 3).

### In vitro assay

Two fistulated Baluchi male sheep were selected for rumen fluid gathering before the morning meal. Sheep were fed a diet containing 60% forage to 40% concentrate based on National Research Council (NRC 2007) recommendations at the maintenance level. The collected rumen fluid was strained through four-layer of cheesecloth and then transferred to the laboratory. The method suggested by Menke and Steingass (1988) was used for the gas test technique. In brief, 200 mg of the milled sample (1 mm screen) was moved into a 100 ml glass syringe. The rumen fluid and artificial saliva at a ratio of 1:2 w/w was added to each syringe under CO_2_ flow and was immediately closed with rubber caps and crimped. All glass syringes were transferred to a water bath at 39 °C. The gas production volume was recorded after 3, 6, 9, 12, 24, 48, 72, and 96 h of incubation. Four replications at two runs were used for each plant species. Also, four glass syringes without samples were selected for blank. Four syringes in each run were separately considered for ruminal fermentation determination. After 24 h incubation, the related syringes were emptied, and their contents filtered through a Buchner funnel assembled with a polyester cloth (45-micron pore size, Kazemi and Ghasemi Bezdi 2021). The content gathered above the polyester cloth was transferred into the pre-weighed crucible, washed with the neutral detergent solution, dried in oven at 60 °C (Makkar 2010) for 48 h, and then true dry matter digestibility (TDMD) was determined. The pH of the in vitro rumen fluid was measured by a pH meter (Hana, Model HI 2210–01, USA) after 24 h of incubation. Ten ml of the strained solution was mixed with 10 ml of 0.2 N HCl and preserved at − 18 °C until ammonia analysis (Komolong et al. 2001). Total protozoa and other genera in the culture medium (24 h incubation) were enumerated using a light microscope and a hemocytometer (Neubauer-improved, Marienfeld, Germany), according to the method described by Dehority (2003). In brief, two mL of each syringe content was pipetted into a screw-capped test tube containing 5 mL of formalized physiological saline (20 mL of formaldehyde in 100 mL of saline containing 0.85 g of NaCl in 100 mL of distilled water). Thereafter, two drops of brilliant green dye solution were added to the test tube, mixed thoroughly, and allowed to stand overnight at room temperature. The method suggested by Getachew et al. (2004) was employed for sampling from rumen fluid and its preparation for ingredients and total volatile fatty acids (TVFA) determination. The volatile fatty acids (VFA) concentrations were determined by gas chromatography (YL6100 GC, Young Lin Instrument, Anyang, South Korea) fitted with a 50 m Silica-fused (0.32 mm internal diameter) column chromatography (CP-Wax Chrom-pack Capillary Column, Varian, Palo Alto, CA, USA). Helium was used as a gas carrier. Crotonic acid was used as an internal standard. The initial and final oven temperatures were set at 55 and 195° C, respectively. The temperature of the detector and injector was set at 250 °C. The data gathered from the gas test were analyzed using the equation $P=b\left(1-{e}^{-ct}\right),$ in which *P* is the volume of gas produced at time *t*, *b* the potential gas production after 96 h incubation (b_gas_, ml/200 mg DM), *c* the fractional rate of gas production (c_gas_, %/h), and *t* the incubation time (h) (Ørskov and McDonald 1979).

### Statistical analysis

The chemical-mineral and buffering capacity were replicated in quadruplicate. The data relating to the gas test were repeated in two runs (four replicates in each run). All data were evaluated for their normality using the Shapiro–Wilk test and then were analyzed in a completely randomized design using SAS software (SAS Institute Inc 2002) with the following model: Y_ij_ = μ + T_i_ + e_ij_ where; Y_ij_ = the value of each observation, μ = total mean, Ti = treatment effect and e_ij_ = experimental error. Statistical differences between treatments were determined at *p* < 0.05 using the Duncan test. Each plant species was considered as a treatment.

## Results

### Chemical compound

The chemical composition of some plant species in 2019 and 2020 is presented in Table 1. A different range of chemical composition was observed among the plant species. The DM content of plants was differed from 15.6% for *Borago officinalis* L. to 44.6% for *Teucrium polium* L. Consistently, in both harvest years (2019 and 2020), CP contents in *Medicago sativa* (19.5–20.3%) were found to be among the highest across the experimental plants, followed by *Melissa officinalis* L. (17.5–18.4%) and *Peganum harmala* L. (17.4–18.2%, *p* < 0.0001). The highest content of NDF, ADF, and ADL was observed in *Cichorium intybus* L. (57–58.2%) and *Perovskia abrotanoides* Kar. (38.5–40.1%) and *Cichorium intybus* L. (16.1–17.4%), respectively (*p* < 0.0001). The ash, EE, and non-fiber carbohydrates (NFC) concentrations were highest in *Borago officinalis* L. (14.8–15.9%) *Perovskia abrotanoides* Kar. (5.40–5.67%), and *Ruta graveolens* L. (48.7–50.7%), respectively (*p* < 0.0001).

**Table 1:** Chemical composition (% of dry matter) of some plant species in 2019 and 2020

Plant species	DM		CP		NDF		ADF		ADL		Ash		EE		NFC	
	2019	2020	2019	2020	2019	2020	2019	2020	2019	2020	2019	2020	2019	2020	2019	2020
*Dracocephalum moldavica* L	20.5^f^	21.9^e^	12.7^d^	13.5^d^	33.8^f^	35.2^g^	16.6^e^	17.8^d^	8.45^c^	9.02^e^	11.3^d^	10.5^c^	2.67^de^	3.89^c^	39.3^c^	38.2^c^
*Melissa officinalis* L	22.2^e^	23.6^d^	17.5^b^	18.4^b^	36.7^e^	37.1^f^	15.7^e^	16.6^de^	9.58^c^	10.2^d^	14.0^b^	12.8^b^	2.98^cd^	3.23^c^	28.6^ef^	28.6^d^
*Ruta graveolens* L	19.8^f^	18.8^f^	14.3^c^	15.5^c^	24.1^g^	25.5^h^	15.0^e^	16.2^e^	5.24^d^	5.76^g^	9.40^e^	8.86^d^	1.62^f^	1.66^e^	50.7^a^	48.7^a^
*Perovskia abrotanoides* Kar	35.9^b^	36.3^b^	7.94^g^	9.01^f^	55.1^b^	53.5^b^	40.1^a^	38.5^a^	12.7^b^	11.8^c^	5.89^g^	6.13^f^	5.67^a^	5.40^a^	26.1^f^	25.7^e^
*Cichorium intybus* L	20.0^f^	18.8^f^	6.57^h^	6.28^g^	58.2^a^	57.0^a^	34.5^b^	33.1^b^	17.4^a^	16.1^a^	8.36^f^	7.97^e^	3.43^c^	4.04^b^	22.9^g^	25.4^e^
*Borago officinalis* L	15.6^g^	16.1^g^	10.4^f^	11.5^e^	37.7^e^	41.8^d^	26.0^d^	27.4^c^	12.4^b^	13.0^b^	15.9^a^	14.8^a^	1.42^f^	1.59^e^	34.6^d^	30.4^d^
*Peganum harmala* L	31.8^c^	32.8^c^	17.4^b^	18.2^b^	21.3^h^	22.5^i^	11.1^f^	11.9^f^	6.30^d^	7.13^f^	13.5^c^	12.9^b^	2.23^e^	2.60^cd^	44.7^b^	44.0^b^
*Teucrium polium* L	43.8^a^	44.6^a^	11.9^e^	12.9^d^	46.1^c^	47.3^c^	30.8^c^	32.1^b^	12.6^b^	13.6^b^	5.43^h^	5.04^g^	4.81^b^	5.34^a^	31.1^e^	29.9^d^
*Medicago sativa*	25.3^d^	23.4^d^	19.5^a^	20.3^a^	42.6^d^	39.3^e^	33.3^b^	31.6^b^	8.50^c^	7.92^f^	8.59^f^	9.06^d^	2.37^e^	2.12^de^	26.9^f^	28.9^d^
SEM	1.31	1.36	0.58	0.86	2.32	2.15	1.93	1.76	0.71	0.63	0.48	0.43	0.27	0.25	1.74	1.55
*p-*value	< 0.0001	< 0.0001	< 0.0001	< 0.0001	< 0.0001	< 0.0001	< 0.0001	< 0.0001	< 0.0001	< 0.0001	< 0.0001	< 0.0001	< 0.0001	< 0.0001	< 0.0001	< 0.0001

Means with different letters among the same row indicate significant differences according to the *p*-value presented

NFC was calculated by subtracting CP, NDF, fat, and ash contents from total DM (Sniffen et al. 1992)

*DM (% of fresh weight)* dry matter, *CP* crude protein, *NDF* neutral detergent fiber, *ADF* acid detergent fiber, *ADL* acid detergent lignin, *EE* ether extract, *NFC* non-fiber carbohydrates, *SEM* standard error of the mean

### Mineral compound and buffering capacity

The mineral composition and buffering capacity of some plant species in 2019 and 2020 are presented in Tables 2 and 3, respectively. The contents of calcium and phosphorus were highest in *Peganum harmala* L. (23.5–24.2 g/kg DM) and *Teucrium polium* L. (7.17–7.67 g/kg DM), respectively (*p* < 0.0001). *Borago officinalis* L. exhibited the highest potassium (28.4–28.9 g/kg DM) and zinc (73.3–75.7 mg/kg DM) among other plants (*p* < 0.0001). The contents of manganese (75.3–85.3 mg/kg DM) and iron (323–385 mg/kg DM) were highest in *Melissa officinalis* L. compared to *Medicago sativa* (*p* < 0.0001). The magnesium content varied from 1.66 for *Medicago sativa* to 12.4 g/kg DM for *Dracocephalum moldavica* L. The pH value of plants was different from 4.85 for *Ruta graveolens* L. to 5.82 for *Medicago sativa*. Titratable acidity was in the range of 128 for *Cichorium intybus* L. to 344 mEq×10^−3^ for *Borago officinalis* L. The highest values related to acid-buffering capacity (189–199 mEq×10^−3^) and acid–base buffering capacity (235–242 mEq×10^−3^) was observed in *Dracocephalum moldavica* L. (*p* < 0.0001). Titratable alkalinity (205–224 mEq×10^−3^) was highest in *Ruta graveolens* L. (*p* < 0.0001). *Medicago sativa* exhibited the highest base-buffering capacity (55.7–58.8 mEq×10^-3^, *p* < 0.0001).

**Table 2:** Mineral composition of some range plant species in 2019 and 2020

Plant species	Ca		P		Na		K		Mg		Mn		Fe		Zn	
	2019	2020	2019	2020	2019	2020	2019	2020	2019	2020	2019	2020	2019	2020	2019	2020
*Dracocephalum moldavica* L	11.6^c^	12.3^b^	5.00^c^	4.80^c^	1.00^d^	0.94^d^	17.9^e^	17.1^e^	11.5^a^	12.4^a^	47.2^d^	44.1^c^	191^b^	193^bc^	43.1^c^	38.4^c^
*Melissa officinalis* L	1.39^e^	1.46^de^	6.43^ab^	7.50^a^	2.67^c^	2.52^c^	22.7^c^	23.6^c^	9.99^b^	10.33^b^	75.3^a^	85.3^a^	385^a^	323^a^	51.1^b^	48.2^b^
*Ruta graveolens* L	1.25^e^	1.15^e^	1.75^f^	1.68^f^	5.65^b^	5.74^b^	17.6^e^	16.6^e^	4.73^d^	4.90^d^	65.5^c^	73.0^b^	80.2^d^	73.3^e^	24.3^e^	24.0^e^
*Perovskia abrotanoides* Kar	5.07^d^	4.92^c^	1.38^f^	1.03^g^	0.39^f^	0.41^f^	12.6^g^	12.1^g^	2.60^f^	2.73^f^	34.3^f^	31.2^e^	92.3^d^	110^de^	34.6^d^	31.3^d^
*Cichorium intybus* L	2.43^e^	2.33^d^	4.20^d^	4.75^c^	0.72^def^	0.79^d^	19.7^d^	20.1^d^	3.26^e^	3.40^e^	39.1^e^	37.4^d^	67.0^d^	69.3^e^	34.0^d^	32.0^d^
*Borago officinalis* L	4.16^d^	4.44^c^	5.77^b^	6.23^b^	0.68^def^	0.73^de^	28.9^a^	28.4^a^	3.32^e^	3.45^e^	46.5^d^	45.0^c^	146^c^	83.0^e^	73.3^a^	75.7^a^
*Peganum harmala* L	24.2^b^	23.5^a^	4.60^cd^	4.12^d^	15.8^a^	14.7^a^	15.9^f^	14.8^f^	8.29^c^	7.93^c^	69.1^b^	72.3^b^	166^bc^	156^cd^	15.0^f^	13.9^f^
*Teucrium polium* L	13.6^b^	11.8^b^	7.17^a^	7.67^a^	0.47^ef^	0.47^ef^	8.88^h^	8.08^h^	2.41^f^	2.44^f^	33.4^f^	29.7^e^	190^b^	217^b^	46.3^bc^	41.5^c^
*Medicago sativa*	11.9^c^	12.8^b^	2.97^e^	3.22^e^	0.80^e^	0.84^d^	25.5^b^	26.5^b^	1.66^g^	1.83^g^	20.6^g^	22.2^f^	145^c^	125^de^	17.4^f^	20.5^e^
SEM	1.41	1.37	0.38	0.44	0.93	0.87	1.20	1.24	0.67	0.71	3.43	4.16	17.8	16.4	3.42	3.39
*p*-value	< 0.0001	< 0.0001	< 0.0001	< 0.0001	< 0.0001	< 0.0001	< 0.0001	< 0.0001	< 0.0001	< 0.0001	< 0.0001	< 0.0001	< 0.0001	< 0.0001	< 0.0001	< 0.0001

Means with different letters among the same row indicate significant differences according to the *p*-value presented

*Ca* calcium (g/kg DM), *P* phosphorus (g/kg DM), *Na* sodium (g/kg DM), *K* potassium (g/kg DM), *Mg* magnesium (g/kg DM), *Mn* manganese (mg/kg DM), *Fe* iron (mg/kg DM), *Zn* zinc (mg/kg DM), *SEM* standard error of the mean

**Table 3:** Buffering capacity (mEq × 10^–3^) of some plant species in 2019 and 2020

Plant species	pH		Titratable acidity		Acid-buffering capacity		Titratable alkalinity		Base-buffering capacity		Acid–base buffering capacity	
	2019	2020	2019	2020	2019	2020	2019	2020	2019	2020	2019	2020
*Dracocephalum moldavica* L	5.22^f^	5.15^e^	315^b^	299^b^	189^a^	199^a^	176^c^	164^c^	46.7^c^	42.6^c^	235^a^	242^a^
*Melissa officinalis* L	5.72^b^	5.76^a^	277^d^	257^c^	120^e^	117^c^	98.2^f^	107^e^	30.0^ef^	33.0^d^	150^e^	150^d^
*Ruta graveolens* L	4.85^g^	4.88^f^	172^f^	178^d^	142^c^	169.8^b^	224^a^	205^a^	54.0^b^	49.9^b^	196^c^	220^b^
*Perovskia abrotanoides* Kar	5.37^e^	5.43^c^	134^g^	148^e^	59.3^h^	65^d^	95.2^f^	104^e^	26.2^g^	29.3^e^	85.5^g^	94.5^e^
*Cichorium intybus* L	5.42^d^	5.41^c^	140^g^	128^f^	70.4^g^	64.4^d^	115^e^	106^e^	32.1^e^	29.6^e^	102^f^	94.0^e^
*Borago officinalis* L	5.79^a^	5.74^a^	344^a^	285^b^	130^d^	108^c^	126^d^	140^d^	39.3^d^	42.9^c^	169^d^	151^d^
*Peganum harmala* L	5.59^c^	5.63^b^	300^c^	327^a^	150^b^	164^b^	183^b^	164^c^	53.6^b^	48.7^b^	204^b^	212^b^
*Teucrium polium* L	5.23^f^	5.32^d^	133^g^	132^ef^	73.7^g^	73.0^d^	109^e^	114^e^	29.1^f^	31.1^de^	103^f^	104^e^
*Medicago sativa*	5.82^a^	5.75^a^	195^e^	196^d^	107^f^	112^c^	187^b^	179^b^	58.8^a^	55.7^a^	166^d^	168^c^
SEM	0.051	0.048	13.6	12.4	6.82	7.90	7.51	6.01	1.99	1.61	8.23	8.99
*p*-value	< 0.0001	< 0.0001	< 0.0001	< 0.0001	< 0.0001	< 0.0001	< 0.0001	< 0.0001	< 0.0001	< 0.0001	< 0.0001	< 0.0001

Means with different letters among the same row indicate significant differences according to the *p*-value presented

*SEM* standard error of the mean

### Protozoa population

Protozoa population of the culture medium after plant incubation are presented in Table 4. Total protozoa and other protozoa populations of the culture medium were changed when some plants were incubated. *Perovskia abrotanoides* Kar., *Cichorium intybus* L., *Peganum harmala* L., and *Teucrium polium* L. had a decreasing effect on total population, *Entodinium* spec., *Diplodinium* spec., *Epidinium* spec., *Ophryoscolex* spec., *Isotricha* spec., and *Dasytrichia* spec., compared to *Medicago sativa* as control plants. The results indicated that *Perovskia abrotanoides* Kar. had the highest decreasing effect on protozoa populations among the studied plants (*p* < 0.0001).

**Table 4:** Protozoa population (× 10^4^cells/ml) of the culture medium after plant incubation

Plant species	Total protozoa		*Entodinium* spec		*Diplodinium* spec		*Epidinium* spec		*Ophryoscolex* spec		*Isotricha* spec		*Dasytrichia* spec	
	2019	2020	2019	2020	2019	2020	2019	2020	2019	2020	2019	2020	2019	2020
*Dracocephalum moldavica* L	22.0^a^	20.3^abc^	17.7^abc^	17.7^a^	1.67^bc^	1.07^b^	0.96^a^	0.95^abc^	0.33^a^	0.27^a^	0.24^a^	0.21^bc^	0.13^a^	0.11^de^
*Melissa officinalis* L	21.5^a^	21.1^a^	18.4^a^	18.0^a^	1.46^c^	1.52^a^	0.95^a^	0.95^bc^	0.32^a^	0.27^a^	0.24^a^	0.23^a^	0.13^a^	0.14^a^
*Ruta graveolens* L	21.8^a^	21.0^ab^	18.2^a^	18.2^a^	1.98^ab^	1.12^b^	0.94^b^	0.96^a^	0.33^a^	0.27^a^	0.24^a^	0.23^ab^	0.13^a^	0.13^ab^
*Perovskia abrotanoides* Kar	15.6^c^	16.7^d^	14.4^d^	14.6^b^	0.11^f^	0.70^c^	0.69^d^	0.91^bc^	0.15^c^	0.21^b^	0.15^c^	0.19^d^	0.11^b^	0.11^e^
*Cichorium intybus* L	18.3^b^	18.6^c^	16.2^c^	16.1^ab^	0.69^de^	0.99^b^	0.87^c^	0.91^bc^	0.27^b^	0.23^ab^	0.20^b^	0.20^cd^	0.11^b^	0.12^bcde^
*Borago officinalis* L	21.9^a^	20.2^abc^	18.0^ab^	17.2^a^	2.16^a^	1.52^a^	0.98^a^	0.96^ab^	0.34^a^	0.25^ab^	0.25^a^	0.22^abc^	0.14^a^	0.13^abc^
*Peganum harmala* L	18.9^b^	19.1^bc^	16.6^bc^	16.6^ab^	0.74^d^	0.96^b^	0.89^bc^	0.94^abc^	0.28^b^	0.25^ab^	0.21^b^	0.21^abc^	0.11^b^	0.13^abcd^
*Teucrium polium* L	18.0^bc^	18.6^c^	16.2^c^	16.0^ab^	0.35^ef^	1.10^b^	0.88^c^	0.91^c^	0.27^b^	0.24^ab^	0.20^b^	0.20^cd^	0.11^b^	0.12^cde^
*Medicago sativa*	22.6^a^	21.6^a^	18.9^a^	18.4^a^	2.07^a^	1.55^a^	0.96^a^	0.97^a^	0.33^a^	0.27^a^	0.24^a^	0.23^ab^	0.13^a^	0.14^a^
SEM	0.46	0.30	0.27	0.30	0.13	0.053	0.015	0.006	0.010	0.005	0.006	0.003	0.002	0.002
*p*-value	< 0.0001	< 0.0001	< 0.0001	0.021	< 0.0001	< 0.0001	< 0.0001	0.019	< 0.0001	< 0.0001	< 0.0001	< 0.0001	< 0.0001	0.0006

Means with different letters among the same row indicate significant differences according to the *p*-value presented

*SEM* standard error of the mean

### In vitro gas production and fermentation parameters

In vitro gas production, ammonia nitrogen, and TDMD of some plants in a culture medium are presented in Table 5. The highest content of b_gas_ (39.1–39.9 ml/200mgDM) and 24 h gas production (30.5–31 ml/200 mg DM) were observed in *Teucrium polium* L. (*p* < 0.0001). The ammonia nitrogen in the culture medium ranged from 18.5 mg/dl for *Cichorium intybus* L. to 31.7 mg/dl for *Medicago sativa*. The content of TDMD also ranged from 57.2% for *Cichorium intybus* L. to 86.8% for *Peganum harmala* L. The measured TVFA and their ingredients from some plants incubated in the culture medium are presented in Table 6. The concentration of TVFA (51.8 for *Perovskia abrotanoides* Kar. to 64.5 for *Ruta graveolens* L.) was different among the incubated plants. The produced acetate and propionate in the culture medium were different among the plant species.

**Table 5:** In vitro gas production, ammonia nitrogen, and true dry matter digestibility of incubated plants in a culture medium

Plant species	b_gas_		c_gas_		gas12		gas24		gas48		gas72		NH_3_-N		TDMD	
	2019	2020	2019	2020	2019	2020	2019	2020	2019	2020	2019	2020	2019	2020	2019	2020
*Dracocephalum moldavica* L	30.1^cd^	30.3^d^	0.026^f^	0.026^g^	7.77^d^	8.25^d^	11.3^f^	12.5^f^	20.9^d^	21.6^f^	24.3^c^	25.2^e^	24.0^de^	21.0^d^	73.4^d^	70.2^b^
*Melissa officinalis* L	16.6^f^	16.1^g^	0.034^ef^	0.036^f^	5.27^e^	5.90^e^	7.62^g^	8.05^g^	11.7^e^	12.0^g^	14.5^d^	15.2^f^	30.3^ab^	27.3^b^	65.3^e^	66.3^c^
*Ruta graveolens* L	35.8^ab^	36.1^b^	0.064^cd^	0.065^d^	17.5^bc^	18.5^c^	27.7^b^	28.3^b^	33.7^b^	34.2^b^	36.0^b^	36.0^b^	28.5^b^	25.0^c^	82.3^b^	83.0^a^
*Perovskia abrotanoides* Kar	23.7^e^	24.2^f^	0.108^a^	0.106^a^	17.2^bc^	17.3^c^	19.2^d^	20.1^d^	22.3^d^	23.3^e^	25.0^c^	24.8^e^	23.4^e^	21.2^d^	59.0^f^	59.2^d^
*Cichorium intybus* L	33.5^bcd^	33.2^c^	0.072^bc^	0.075^bc^	19.3^b^	20.2^b^	23.8^c^	24.7^c^	29.8^c^	28.5^d^	34.4^b^	33.3^d^	22.2^e^	18.5^e^	57.2^f^	59.2^d^
*Borago officinalis* L	29.4^d^	28.0^e^	0.030^f^	0.034^fg^	8.90^d^	9.47^d^	14.9^e^	15.5^e^	21.1^d^	21.8^ef^	26.7^c^	26.2^e^	26.1^cd^	24.2^c^	74.9^d^	71.2^b^
*Peganum harmala* L	33.8^bc^	33.4^c^	0.086^b^	0.084^b^	22.2^a^	21.4^ab^	27.5^b^	26.2^c^	31.5^bc^	29.5^cd^	33.9^b^	33.8^cd^	31.2^a^	27.3^b^	86.8^a^	81.7^a^
*Teucrium polium* L	39.9^a^	39.1^a^	0.072^bc^	0.071^cd^	21.8^a^	22.4^a^	31.1^a^	30.5^a^	38.3^a^	37.6^a^	40.0^a^	39.7^a^	26.3^c^	27.0^b^	66.1^e^	57.5^c^
*Medicago sativa*	36.0^ab^	36.7^b^	0.050^de^	0.053^e^	16.6^c^	17.2^c^	24.6^c^	25.8^c^	29.0^c^	30.8^c^	34.6^b^	35.5^bc^	30.8^a^	31.7^a^	79.3^c^	81.0^a^
SEM	1.37	1.33	0.005	0.005	1.18	1.14	1.51	1.44	1.53	1.44	1.48	1.43	0.66	0.77	1.93	1.71
*p*-value	< 0.0001	< 0.0001	< 0.0001	< 0.0001	< 0.0001	< 0.0001	< 0.0001	< 0.0001	< 0.0001	< 0.0001	< 0.0001	< 0.0001	< 0.0001	< 0.0001	< 0.0001	< 0.0001

Means with different letters among the same row indicate significant differences according to the *p*-value presented

*b*_*gas*_ potential gas production (ml/200 mg DM), *c*_*gas*_ fractional rate of gas production (%/h), *gas 12, 24, 48, and 72 h* cumulative gas production after 12, 24, 48, and 72 h incubation (ml/200 mg DM), *NH*_*3*_*-N* ammonia nitrogen (mg/dl), *TDMD* true dry matter digestibility (%, 24 h incubation), *SEM* standard error of the mean

**Table 6:** The measured total volatile fatty acids (mmol/l) and their ingredients (% of TVFA) from some plants incubated in the culture medium after 24 h incubation

Plant species	TVFA		Acetate		Propionate		Butyrate		Valerate		iso-Valerate		pH24h	
	2019	2020	2019	2020	2019	2020	2019	2020	2019	2020	2019	2020	2019	2020
*Dracocephalum moldavica* L	58.1^d^	53.7^d^	63.3^ab^	63.0^a^	19.8^b^	19.7^abc^	13.9	14.0	1.25	1.20	0.40	0.38	6.90^a^	6.87^a^
*Melissa officinalis* L	61.5^b^	56.9^c^	64.0^a^	62.8^a^	19.3^b^	18.0^c^	13.8	14.7	1.20	1.30	0.45	0.38	6.92^a^	6.91^a^
*Ruta graveolens* L	64.5^a^	60.5^ab^	60.5^c^	60.8^c^	23.7^a^	21.4^a^	13.2	14.0	1.38	1.40	0.41	0.42	6.89^a^	6.86^ab^
*Perovskia abrotanoides* Kar	56.6^e^	51.8^e^	63.5^ab^	63.0^a^	19.8^b^	18.7^bc^	13.5	14.6	1.22	1.32	0.45	0.43	6.91^a^	6.85^ab^
*Cichorium intybus* L	63.5^a^	59.1^b^	61.6^bc^	63.1^a^	22.7^a^	21.0^ab^	13.5	13.7	1.33	1.33	0.41	0.38	6.89^a^	6.88^a^
*Borago officinalis* L	59.7^c^	55.0^d^	62.0^abc^	61.2^bc^	22.8^a^	21.7^a^	13.3	13.7	1.35	1.40	0.41	0.41	6.92^a^	6.90^a^
*Peganum harmala* L	63.8^a^	60.0^ab^	63.3^ab^	62.2^ab^	18.2^b^	18.3^c^	14.5	15.0	1.47	1.40	0.49	0.48	6.89^a^	6.86^ab^
*Teucrium polium* L	64.0^a^	60.7^a^	62.7^ab^	63.1^a^	19.9^b^	19.2^abc^	13.6	14.3	1.35	1.22	0.38	0.45	6.82^b^	6.80^b^
*Medicago sativa*	63.9^a^	59.9^ab^	62.2^abc^	62.5^a^	19.7^b^	20.2^abc^	14.2	14.6	1.42	1.26	0.42	0.49	6.89^a^	6.86^ab^
SEM	0.55	0.62	0.27	0.19	0.40	0.33	0.14	0.16	0.028	0.024	0.014	0.014	0.0076	0.0078
*p*-value	< 0.0001	< 0.0001	0.026	0.001	0.0004	< 0.0001	0.52	0.44	0.36	0.479	0.72	0.34	0.05	0.0006

Means with different letters among the same row indicate significant differences according to the *p*-value presented

*TVFA* total volatile fatty acids, *SEM* standard error of the mean

## Discussion

Despite the increasing scientific interest in range plants as forage feed, the chemical composition and nutritional properties of some range plants have not yet been commonly studied. We decided to evaluate the nutritional properties of eight plant species of different genera compared to alfalfa. As expected, the overall variation in the chemical composition of plant species between 2019 and 2020 was low. *Melissa officinalis* L. (17.5–18.4%) and *Peganum harmala* L. (17.4–18.2%) were rich sources of CP compared to *Medicago sativa*. In contrast with our study, the CP content of different *Melissa officinalis* L. samples was reported to be 4.14–7.74% DM (Dias et al. 2012). In our study, a higher fat content for *Melissa officinalis* L. was observed compared to the previous study (Dias et al. 2012). The range of NDF (21.3–58.2%), ADF (11.1–40.1%), and ADL (5.24–17.4%) concentrations of present plants are in agreement with other reports (Kazemi 2019; Kazemi and Valizadeh 2019). Kazemi (2019) reported that the chemical composition of plants can alter in different weather and different harvesting time. Considering a minimum level of CP (7–8% of DM) required for a balanced rumen function and dry matter intake (DMI) in small ruminants, the concentration of CP in the present plants can be helpful in this regard (Van Soest 1994). The higher content of NDF in *Cichorium intybus* L. can be attributed to the lower leaf to stem ratio. Part of the high levels of fat in *Perovskia abrotanoides* Kar and *Teucrium polium* L. can be attributed to the high amounts of essential oils that have been previously reported by researchers (Ghaffari et al. 2018; Mahmoudi and Nosratpour 2013). The forage digestibility and DMI in ruminants are influenced mainly by the dietary NDF (Harper and McNeill 2015). We found a reasonable range of NDF among the present plants. Feeds and forages like of present plants are extensively different in their amount and composition of NFC, and carbohydrates fractions in NFC vary in rate and extent of ruminal fermentation, fermentation products, and contribution in microbial yield (Hall and Herejk 2001; Nocek and Tamminga 1991), and therefore in ruminants performance.

Forages supply a significant source of minerals for livestock. The bioavailability of forage minerals to ruminants may be affected by the distribution of minerals within the forage and the chemical form of the elements present (Spears 1994; Khan et al. 2005). In the present study, we found a diverse range of minerals among the studied plants. Awareness of mineral requirements and the bioavailability of forages will allow animal nutritionists to formulate mineral supplements that maximize animal performance. Therefore, the reported data about the mineral contents of the present plants (Table 2) can be helpful for nutritionists in preparing a balanced diet. In the present study, the level of potassium in all the plants was above the critical levels (6–8 g/kg DM) recommended for grazing animals (McDowell 1992, 1997). However, it has been reported that high-yielding animals under heat stress require potassium above 10 g/kg DM (Mirzaei 2012). The present plants contained suitable levels of Fe (67–385 mg/kg DM) as the recommended level of iron for ruminants was 30 mg/kg DM (McDowell 1985). It is reported that the forage manganese level between 30 and 40 mg/kg can meet the manganese requirements of ruminants (McDowell 1985). In this study, except *Medicago sativa*, the manganese content of other plants was above 30 mg/kg DM. A recommended sodium requirement for grazing ruminants has been reported between 0.4 and 1.8 g/kg DM (McDowell 1985). We found that all studied plants had a Na content above 0.4 g/kg DM, and they will be able to meet the sodium requirements of ruminants quickly. The critical dietary level of phosphorus concentration is 2.5 g/kg DM (McDowell 1992, 1997), and it is reported that the phosphorus requirement of a grazing ruminant is rarely met by forages. So supplementation of 1 kg of diets (DM basis) with *Dracocephalum moldavica* L., *Melissa officinalis* L., *Cichorium intybus* L., *Borago officinalis* L., *Peganum harmala* L., *Teucrium polium* L., and *Medicago sativa* can meet the phosphorus requirements of ruminants. Calcium and phosphorus play crucial roles in most body tissues with structural functions in cell membranes, bones, and teeth (McDowell and Arthington 2005). They are involved in cellular processes including membrane potential, DNA synthesis, cell membrane fluidity, intracellular communication, and biochemical pathways (Underwood and Suttle 1999). Minson (1990) reported that among the 263 samples, only 31% of forages were low in calcium (less than 3 g/kg DM). We found that only *Melissa officinalis* L., *Ruta graveolens* L., and *Cichorium intybus* L. had calcium content below critical concentrations (3 g/kg DM). Except for *Medicago sativa*, the magnesium concentration of all present plants is well more than the critical level (2 g/kg DM; McDowell 1992, 1997) for grazing animals. The critical level of zinc for ruminants has been reported at about 30 mg/kg DM (McDowell 1985). The zinc concentrations of *Dracocephalum moldavica* L., *Melissa officinalis* L., *Perovskia abrotanoides* Kar., *Cichorium intybus* L., *Borago officinalis* L., and *Teucrium polium* L. is more than the critical level (i.e., 38.4–43.1, 48.2–51.1, 31.3–34.6, 32–34, 73.3–75.7, and 41.5–46.3 mg/kg DM, respectively), and those of *Ruta graveolens* L., *Peganum harmala* L., and *Medicago sativa* are less than the critical level (i.e., 24–24.3, 13.9–15, 17.4–20.5 mg/kg DM, respectively) (Table 2).

Three significant mechanisms, including the salivary buffer system, the buffering capacity of the feed consumed, and the dietary additive buffers, can affect the ruminal buffering system in ruminants (Moharrery 2007). Initial pH and titratable acidity have been reported to be the most critical determinants of rumen fluid pH. In the present study, the highest titratable acidity was observed for *Borago officinalis* L. (285–344 mEq × 10^–3^) and *Peganum harmala* L. (300–327 mEq × 10^–3^), indicating high resistance to acidification. Due to the different ash content of the plant species studied in this study (5.89–15.9%), their buffering capacity was also different. In line with our study, the buffering capacity of some protein sources and leguminous fodder has been reported higher than 85 mEq × 10^–3^ (Montañez-Valdez et al. 2013). By evaluating the pH and buffering capacity of the diet, we can predict the need for buffers to control and maintain rumen pH (Bujňák et al. 2011). Except for *Ruta graveolens* L., other plants had near-neutral pH, and therefore, their consumption could not lead to rumen pH reduction. It is reported that the amount and composition of minerals in the ash have a particular buffering effect on the plant’s initial pH (Levic et al. 2005). In this study, the highest acid and also acid–base buffering capacity in *Dracocephalum moldavica* L. indicated more acid is needed to change in pH of the water-soluble plant sample and high control of this plant in ruminal pH balance.

Even though protozoa include a considerable section of the rumen biomass, their function in ruminal fermentation and their contribution to the metabolism and nutrition of the animal is still an area of substantial controversy (Williams and Coleman 1992). In the present study, we found that the plants including *Perovskia abrotanoides* Kar., *Peganum harmala* L., and *Teucrium polium* L. had an antiprotozoal activity in the culture medium compared to the *Medicago sativa*. Wright and Phillipson (1990) also reported an antiprotozoal activity of *Peganum harmala* L. because of its alkaloid compounds. It is reported that *Perovskia* is a small genus from the *Lamiaceae* family, which includes a variety of promising medicinal and phytochemical properties (Mohammadhosseini et al. 2019). In line with the present study, an anti-protozoan activity by n-hexane and ethyl acetate extracts from the aerial parts of *Perovskia* was reported by Tabefam et al. (2018). The antimicrobial effect of alcoholic extracts of *Teucrium polium* L. was reported by Darabpour et al. (2010). It is reported that some plants such as *Teucrium polium* L. can produce some antimicrobial substances in themselves (Darabpour et al. 2010). Protozoa predate on bacteria as a major source of protein (Williams and Coleman 1992) and as a result, defaunation makes the rumen more efficient in terms of proteosynthesis, increasing the duodenal microbial protein flow and total non-ammonia nitrogen flow. Therefore, it seems that the reduction of the protozoan population due to incubation of *Perovskia abrotanoides* Kar., *Peganum harmala* L., and *Teucrium polium* L. is effective in improvement of animal performance.

When a diet is incubated with buffered rumen fluid in a culture medium, the carbohydrates are mainly fermented to SCFA, microbial cells, and gases (mainly CH_4_ and CO_2_) (Getachew et al. 1998). Gas production is basically a result of carbohydrates fermentation to acetate, propionate, and butyrate (Beuvink and Spoelstra 1992; Blümmel and Ørskov 1993). We found that *Teucrium polium* L. had higher potential gas production. Although the increase in gas production due to incubation of *Teucrium polium* L. can indicate more fermentation in vitro, more gas production cannot always indicate higher quality of forages or be associated with more digestibility because the gas produced in the gas technique is the direct gas produced as a result of fermentation (CO_2_ and CH_4_) and the indirect gas produced from the buffering of short chain fatty acids (CO_2_ released from the bicarbonate buffer) (Getachew et al. 1998). Several factors such anaerobiosis, proper temperature, suitable pH, and adequate buffering affect the fermentation of feedstuffs by ruminal microorganisms and hence gas production (Getachew et al. 1998). Browse plants and tree fodders contain a valuable source of nutrients for ruminants worldwide. Although these plants can be considered as good sources of valuable nutrients, they often contain different amounts of secondary compositions. Such secondary compositions are known to affect nutrient utilization due to their interaction with nutrients, and also because they influenced ruminal microorganisms (Getachew et al. 1998). Although different secondary compounds were reported for *Teucrium polium* L. as a medicinal plant (Farahbakhsh et al. 2020; Al-Otaibi and AlMotwaa 2021), the reasons for increased gas production here are unclear for us. A section of increase in potential gas production resulting from *Teucrium polium* L. incubation may be attributed to its secondary compound. We found that *Ruta graveolens* L., *Cichorium intybus* L., and *Peganum harmala* L. produced a potential gas production almost similar to *Medicago sativa*. It has been reported that gas production is a reflection of generation of short chain fatty acids (Getachew et al. 1998).

We found a normal pH (6.80–6.92) in the culture medium when plants were incubated. The reported values of pH (Table 6) are within the normal pH (5.8–7.2) that has been reported by Hiltner and Dehority (1983) for favorite ruminal microbial activity. The VFA produced via ruminal microbial fermentation are the primary source of energy absorbed by the digestive tract wall of ruminants and they can affect on animal performance. The efficiency of energy utilization, methane yield, risks of ruminal acidosis, and composition of animal products can be affected by the profile and concentration of VFA (Noziere et al. 2011). In this study, TVFA produced from *Ruta graveolens* L., *Cichorium intybus* L., *Peganum harmala* L., and *Teucrium polium* L. incubation was approximately similar to *Medicago sativa* in the culture medium. It has been reported that up to 80% of maintenance energy required for ruminants is supplied by VFA (Bergman 1990; Baldwin 1998). Therefore, the production of more VFA due to incubation of *Ruta graveolens* L., *Cichorium intybus* L., *Peganum harmala* L., and *Teucrium polium* L. can be beneficial to the host animal to meet energy requirements. We found that among the studied plants, only, *Ruta graveolens* L. produced less acetate after incubation. Propionate is an important gluconeogenic substrate for ruminants, whereas acetate is the primary precursor for de novo lipogenesis (Ötztürk et al. 2015). All plants produced suitable propionate concentrations compared to the *Medicago sativa*.

In summary, many rangeland plant species provide a suitable reserve feed for grazing animals, particularly during the dry season, or fill regular gaps in feed supply caused by seasonal conditions. The data about the chemical-mineral compounds, buffering capacity, in vitro gas production, and in vitro ruminal microbial fermentation characteristics of eight rangeland plants obtained under well situation and similar experimental conditions can be used to optimize diet formulation in terms of nutrients supply to the small ruminants that will ensure better utilization of this nutrients-rich feed resource and will be profitable from an economic point of view for animal husbandries in this region and other similar regions worldwide. *Melissa officinalis* L. and *Peganum harmala* L. were found to be a suitable source of CP compared to *Medicago sativa*. The calcium content of *Peganum harmala* L. was approximately two times higher than *Medicago sativa*. *Dracocephalum moldavica* L. exhibited higher acid–base buffering capacity compared to other plants. More potential gas production was observed in *Teucrium polium* L. The plants, including *Perovskia abrotanoides* Kar., *Cichorium intybus* L., *Peganum harmala* L., and *Teucrium polium* L. had anti-protozoan activity. Based on previous reports, *Peganum harmala* L. might be toxic for animals if it is consumed excessively. Therefore, dietary consumption of this plant should be controlled, especially in younger animals. Variation in the studied parameters among the investigated forages between 2019 and 2020 was negligible. Overall, these results indicate that plant species have relatively high nutritional values hence they can be used as sources of feeds for livestock to improve their production performance.

## Data Availability

The data will be made available upon request.

## Abbreviations

DM: Dry matter
EE: Ether extract
CP: Crude protein
NDF: Neutral detergent fiber
ADL: Acid detergent lignin
ADF: Acid detergent fiber
NRC: National research council
TDMD: True dry matter digestibility
TVFA: Total volatile fatty acids
VFA: Volatile fatty acids
b_gas_: Potential gas production
c_gas_: Fractional rate of gas production
NFC: Non-fiber carbohydrates
DMI: Dry matter intake
